# Supplementary material for: Phosphate (Pi) Transporter PIT1 Induces Pi Starvation in Salmonella-Containing Vacuole in HeLa Cells
Source: Int J Mol Sci. 2023 Dec 7;24(24):17216. doi: 10.3390/ijms242417216 (PMC10743064; doi:10.3390/ijms242417216)
Supplement: Supplementary file 1 [file ijms-24-17216-s001.zip › ijms-2716905-supplementary.pdf]

Supplementary Figures

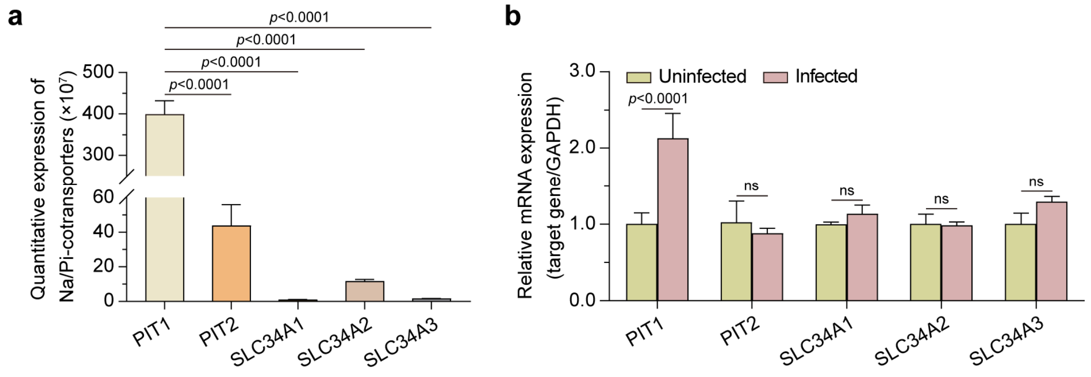

Figure S1. *S.* Typhimurium infection upregulates PIT1 expression in HeLa cells. (**a**) Quantitative PCR analysis of the amount of SLC20 (PIT1 and PIT2) and SLC34 (SLC34A1, SLC34A2, and SLC34A3) in HeLa cells. Data indicate means ± SD (n = 3). The significant differences are represented by p-values determined with one-way ANOVA. (**b**) qRT-PCR analysis of the relative mRNA expression of SLC20 (PIT1 and PIT2) and SLC34 (SLC34A1, SLC34A2, and SLC34A3) at 2 h p.i. in HeLa cells. Data indicate means ± SD (n = 3). The significant differences are represented by p-values determined with two-way ANOVA. ns, nonsignificant.

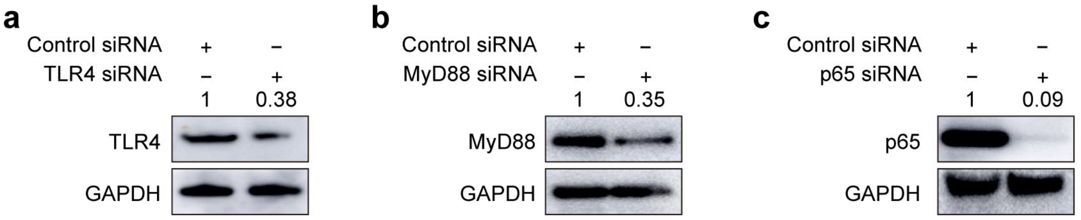

Figure S2. The transfection efficiency of TLR4, MyD88, or p65 siRNA in HeLa cells. (**a**) Western blotting analysis to show the silencing efficiency of TLR4 siRNA in HeLa cells. HeLa cells were transfected with control siRNA or TLR4 siRNA. (**b**) Western blotting analysis to show the silencing efficiency of MyD88 siRNA in HeLa cells. HeLa cells were transfected with control siRNA or MyD88 siRNA. (**c**) Western blotting analysis to show the silencing efficiency of p65 siRNA in HeLa cells. HeLa cells were transfected with control siRNA or p65 siRNA. The silencing efficiency is indicated at the top of the blots.

Supplementary Tables

Table S1. Strains and plasmids used in this study.

Strains	Genotype or description	Source or reference
S. Typhimurium WT	Wild-type *Salmonella enterica* serovar Typhimurium strain, ATCC 14028	ATCC*
Δ*msbB*	*S*. Typhimurium with deletion of *msbB*	This work
Δ*phoBR*	*S*. Typhimurium with deletion of *phoBR*	This work
S. Typhimurium-GEP	*S*. Typhimurium containing pETDuet-GFP	This work
Δ*msbB*-GFP	Δ*msbB* containing pETDuet-GFP	This work
**Plasmids**		
pSIM17	Red recombination plasmid, Blastidin	Gene R, 2006
pKD3	Containing a chloramphenicol resistance cassette and the flipase recognition sites, Chl	Datsenko KA, 2000
pKD4	Containing a kanamycin resistance cassette and the flipase recognition sites, Kan	Datsenko KA, 2000
pcDNA3.1	Mammalian expression vector, Amp	Invitrogen
pcDNA3.1-PIT1	pcDNA3.1 carrying ORF of PIT1, Amp	This work
pETDuet-GFP	pETDuet carrying fragment of GFP, Amp	This work

*, ATCC, American Type Culture Collection, Manassas, Virginia, USA.

Table S2 Primers used in this study (5'-3').

**Primers for gene mutation**		
msbB	F	GGTTTGTCAGCATAAAGCCTCTCTTACGAGAGGCTTTATGTGTAGGCTGGAGCTGCTTC
msbB	R	TTTCGCGTCAGCAGACCCTGGAAAAGCATGGAAACCAAAATGGGAATTAGCCATGGTCC
phoBR	F	TGGCGCGGCATTGATAACTAACGACTAACAGGGCAAATTGTGTAGGCTGGAGCTGCTTC
phoBR	R	TGGCGGAGAAGGCTTTCCATTCTTGAGGGAGTATGACGCGTGTAGGCTGGAGCTGCTTC
**Primers for gene identification**		
msbB	F	GCCATGTCTGCGATGTATGC
msbB	R	GGTGAAACCGGGGCAAAAAG
phoBR	F	CCAGATGCCAGTCAGAGGTG
phoBR	R	CAGCACCTGAAGCGCAATTT
**Primers for pcDNA3.1-PIT1**		
pcDNA3.1-PIT1	F	GGAAGATCTATGATGGCAACGCTGATTACC
pcDNA3.1-PIT1	R	CCGCTCGAGGCATTCTGAGGATGACATATC
**Primers for qRT-PCR**		
PIT1	F	GTTCGTGCATTCATCCTCCAT
PIT1	R	TGGTACCCCACAGAGGAAGTTT
PIT2	F	TCTCATGGCTGGGGAAGTTAGT
PIT2	R	TTGCGACCAGTGAGAATCCTAT
SLC34A1	F	TCACTTTCATCCCCTTTCTGG
SLC34A1	R	GGCGTCAATGGGAACAGG
SLC34A2	F	CAGGAGTCGGAGGCACAGTA
SLC34A2	R	TGCCAAGTATCGCTGGTTCG
SLC34A3	F	GACTGGTCATTGGCGTGC
SLC34A3	R	GCTGGTGATGGATGTGCCTA
GAPDH	F	AGCAATGCCTCCTGCACCACCAAC
GAPDH	R	CCGGAGGGGCCATCCACAGTCT
ssrA	F	CTGGACCTCTTGCTGGCTGAT
ssrA	R	TGGCGTAAGTCGGTTAGTTCCT
ssrB	F	AAGTTCTGTTAGCGGCATTGC
ssrB	R	AGCAGTTGATGATTGGTCGTGT
sifA	F	ATGATGCCACCATTATTCTTCG
sifA	R	CGTCATTTGTGGATGCGATT
16S	F	GAAAGCGTGGGGAGCAAAC
16S	R	ACATGCTCCACCGCTTGTG
